# Acute Liver Failure Etiology Determines Long-Term Outcomes in Patients Undergoing Liver Transplantation: An Analysis of the UNOS Database

**DOI:** 10.3390/jcm13226642

**Published:** 2024-11-05

**Authors:** Natalia Rojas Amaris, Ana Marenco-Flores, Romelia Barba, Denisse Rubio-Cruz, Esli Medina-Morales, Daniela Goyes, Behnam Saberi, Vilas Patwardhan, Alan Bonder

**Affiliations:** 1 Division of Gastroenterology, Hepatology, and Nutrition, Beth Israel Deaconess Medical Center, Harvard Medical School, Boston, MA 02215, USA; nrojasam@bidmc.harvard.edu (N.R.A.); amarenco@bidmc.harvard.edu (A.M.-F.); drubiocr@bidmc.harvard.edu (D.R.-C.); bsaberi@bidmc.harvard.edu (B.S.); vpatward@bidmc.harvard.edu (V.P.); 2 Department of Internal Medicine, Texas Tech University System, Lubbock, TX 79430, USA; romelia.barba@ttuhsc.edu; 3 Department of Medicine, Rutgers New Jersey Medical School, Newark, NJ 07103, USA; jm2831@njms.rutgers.edu; 4 Division of Digestive Diseases, Yale School of Medicine, New Haven, CT 06520, USA; daniela.goyes@yale.edu

**Keywords:** acute liver failure, waitlist mortality, post-transplant mortality

## Abstract

**Background**: Acute liver failure (ALF) involves rapid liver injury, often leading to multi-organ failure. Liver transplantation (LT) has improved survival rates, with U.S. rates reaching 92%. This study analyzes UNOS data (2002–2020) to evaluate long-term survival and identify risk factors affecting waitlist and post-LT outcomes in ALF patients. **Methods**: A retrospective analysis was performed on adult ALF patients waitlisted for LT (Status 1/1A). ALF etiologies, including viral infections, drug-induced liver injury (DILI), acetaminophen (APAP) overdose, autoimmune hepatitis (AIH), Wilson disease (WD), and unknown causes, were assessed with patient and donor characteristics. Kaplan–Meier and Cox regression analyses identified predictors of patient and graft survival. Sensitivity analysis confirmed the model’s robustness. **Results**: We identified 2759 ALF patients. APAP (HR 1.7; *p* < 0.001) and unknown etiology (HR 1.3; *p* = 0.037) were linked to higher waitlist removal risk, while WD (HR 0.36; *p* < 0.001) increased LT probability. Among 2014 LT recipients, WD showed improved survival (HR 0.53; *p* = 0.002). Black/African American race (HR 1.47; *p* < 0.001), diabetes (HR 1.81; *p* < 0.001), and encephalopathy (HR 1.27; *p* < 0.001) predicted higher mortality. AIH had the lowest 1- and 10-year survival (83% and 62%), while APAP had the lowest 5-year survival (76%). WD had the highest graft survival at 1, 5, and 10 years (93%, 88%, and 80%). **Conclusions**: ALF etiology significantly affects survival outcomes. AIH and APAP are associated with worse survival, while WD shows favorable outcomes. Tailored post-LT management is essential to improve survival in ALF patients.

## 1. Introduction

Acute liver failure (ALF) is characterized by the rapid onset of severe hepatic injury, resulting in coagulopathy (INR > 1.5) and hepatic encephalopathy (HE), often progressing to multi-organ failure, all occurring in the absence of pre-existing liver disease [1]. The definition of ‘acute’ varies globally, with time frames ranging from 4 weeks in India to 26 weeks in North America and up to 28 weeks in Europe [2]. ALF has an incidence of approximately 1.61 cases per 100,000 persons annually, with higher rates reported in Asian populations, reaching 23.8 per 100,000 per year. It is most commonly observed in women between the ages of 20 and 40 [3].

Over the past two decades, outcomes for patients with ALF, both with and without liver transplantation (LT), have steadily improved. Two decades ago, ALF was associated with mortality rates as high as 85% [4]. However, with the adoption and maturation of LT as a therapeutic option, mortality rates have decreased significantly, with current 1-year survival rates reported at 74% in Europe and up to 92% in the United States (U.S.) [3,4,5]. Data from the European Liver Transplant Registry (1988–2009) and the U.S. registries, the Acute Liver Failure Study Group (ALFSG) and Scientific Registry of Transplant Recipients (SRTR) (1998–2018), respectively [6], support these findings. ALF now accounts for nearly 10% of the indications for LT worldwide [3,5].

The diverse etiologies of ALF contribute significantly to the variability in patient outcomes [7]. Data from the ALFSG indicate that drug-induced liver injury (DILI) accounts for over 50% of ALF cases in the United States [7,8]. Other causes of ALF included viral hepatitis (hepatitis A, B, E, and non-hepatotropic viruses), acute presentations of Wilson disease (WD), autoimmune hepatitis (AIH), and Budd–Chiari syndrome [2,9,10]. Tujios et al. classified ALF etiologies into those associated with favorable and unfavorable outcomes [8]. Despite this, comparative outcomes across these etiological subgroups remain insufficiently studied, and long-term outcomes have not been reported extensively. Further exploration of the outcomes associated with specific ALF subgroups is necessary to refine clinical decision-making and improve patient prognosis.

The impact of etiology on post-LT outcomes in ALF remains poorly characterized, with most studies primarily focusing on short- to mid-term survival rather than long-term outcomes. In this study, we conducted a retrospective analysis of data from patients listed for LT due to ALF in the U.S. between 2002 and 2020. The data were sourced from the United Network for Organ Sharing (UNOS) database. The primary objective is to evaluate the impact of ALF etiology on LT waitlist outcomes among patients listed for LT in the U.S. The secondary aim is to assess long-term patient and graft survival following LT for ALF, focusing on outcome variations based on etiology. Additionally, we aim to identify potential risk factors for post-transplant death or graft loss and explore whether these factors could help predict poor prognoses.

## 2. Materials and Methods

### 2.1. Study Population

We conducted a retrospective cohort study using the UNOS database to identify adult patients listed for LT due to ALF between January 2002 and December 2020, classified as Status 1 or 1A. Patients with Status 1/1A have ALF and a projected survival of less than seven days without LT. Status 1B candidates were excluded from this study, as they were under 18 years of age and had underlying chronic liver disease. According to UNOS criteria, adults with ALF are eligible for Status 1 listing if they have no preexisting liver disease, except for WD, and are admitted to an intensive care unit (ICU) with one or more of the following conditions: (1) ventilator dependence, (2) dialysis dependence, or (3) an INR > 2.0.

**Figure 1:**
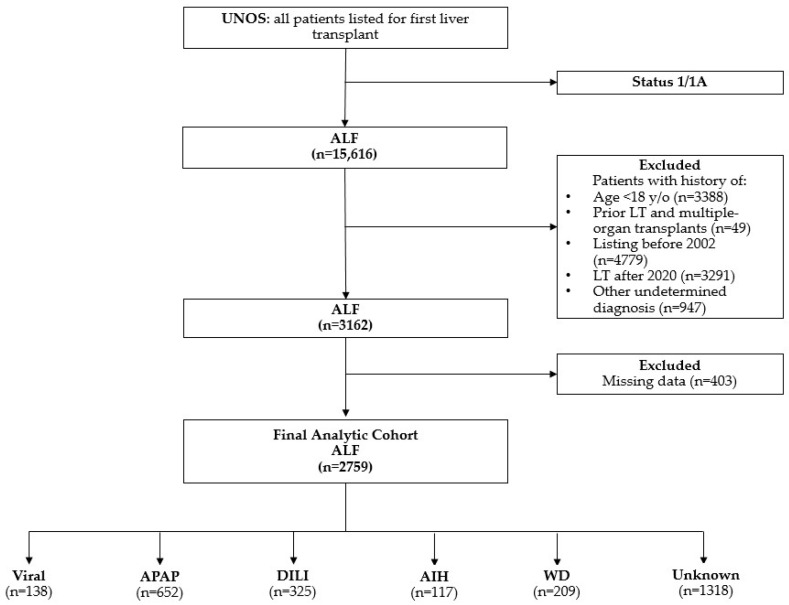
Flowchart of exclusion criteria. AIH, autoimmune hepatitis; ALF, acute liver failure; APAP, acetaminophen; DILI, drug-induced liver injury; LT, liver transplant; viral: hepatitis A and B viruses; WD, Wilson disease; y/o, years old.

Our analysis included patients waitlisted to receive an LT under Status 1/A1. We excluded patients who (1) were under 18 years of age at the time of listing, (2) were listed for multiple organ transplants, or (3) had a history of prior LT (Figure 1). Patients were categorized by the etiology of ALF, including viral-related causes (hepatitis A [HAV], hepatitis B [HBV]), DILI, acetaminophen (APAP)-induced liver injury, AIH, WD, and unknown causes. At the time of waitlisting, etiology was determined from UNOS coding and free-text entries. If no clear etiology was identified or if the coding/text was nonspecific, the patient was classified as having an unknown etiology. The “unknown etiology” category was defined using specific UNOS database codes, including 999 and 4108 (unknown), 4268 and 4213 (cryptogenic cirrhosis), and 4209 (unknown cirrhosis). This category includes patients with either a missing etiology (potentially due to administrative limitations), an explicit “unknown” label, or insufficient detail in free-text entries to assign a specific etiology. The classification of APAP versus other DILI cases relied solely on free-text entries due to the absence of specific UNOS codes.

### 2.2. Study Outcome, Variables, and Definitions

The primary outcome of this study was to evaluate the impact of ALF etiology on waitlist survival. Waitlist survival was defined as the composite outcome of removal due to death, clinical deterioration, or clinical improvement (UNOS removal codes 8, 12, and 13). Secondary outcomes included patient and graft survival following LT. Patient survival was defined as the time from the date of transplantation to either the recipient’s death or the last follow-up if the patient was lost to follow-up. Graft survival was defined as the time from the transplant to graft failure or the need for a repeat LT.

Patient characteristics were compared based on different etiologies of liver disease. Since recipient and donor characteristics varied, we analyzed them separately. For recipients, we assessed a comprehensive set of variables, including age at listing for transplant, gender, self-reported race/ethnicity, college or university education, type of insurance, U.S. citizenship, blood type, body mass index (BMI), history of diabetes mellitus (DM), laboratory values (such as sodium, serum creatinine, and INR), Model for End-Stage Liver Disease (MELD) score at listing, presence of moderate-to-severe ascites, grade 3–4 encephalopathy, and waiting time for transplantation (in days). For donors, we evaluated quality parameters, including donor age, gender, BMI, and cold ischemia time (in hours).

The authors are solely responsible for analyzing and interpreting these data, and the opinions expressed in this study do not reflect the official stance of the Organ Procurement and Transplantation Network (OPTN) or the U.S. Government. Since UNOS provides a publicly accessible, de-identified patient-level database, institutional review board (IRB) approval was not required by the policies of both UNOS and Beth Israel Deaconess Medical Center.

### 2.3. Statistical Analysis

Clinical and demographic characteristics were stratified by the etiology of ALF and compared cohort characteristics using the Kruskal–Wallis test for continuous variables and Pearson’s chi-squared test (χ^2^) for categorical variables. Continuous variables were reported as medians with interquartile ranges (IQR), and categorical variables were summarized using percentages.

We conducted Kaplan–Meier survival analyses to estimate survival outcomes and used the log-rank test to assess significant differences between groups. To identify critical predictors of survival, we applied forward stepwise multivariate Cox regression analyses, adjusting for both recipient and donor characteristics. Variables that were statistically significant at the bivariate level (entry and removal thresholds set at 0.1 and 0.05, respectively) or considered clinically important were included in the model. Recipient factors included age at transplant, gender, race, college or university education, public insurance, U.S. citizenship, blood type, BMI, diabetes status, sodium levels, serum creatinine, MELD score, bilirubin, presence of ascites, encephalopathy, and wait time. Donor factors included age, gender, BMI, and cold ischemia time. Results are reported as hazard ratios (HR) with 95% confidence intervals (CIs), with statistical significance defined at α = 0.05. Patients with viral-related infections (specifically HAV and HBV) served as the reference group.

To assess the robustness of our model’s associations, we conducted an E-value sensitivity analysis to evaluate the impact of potential unmeasured confounding. The E-value indicates the minimum association strength an unmeasured confounder would need with both the exposure and outcome to explain an observed association. We calculated E-values for HRs from Cox regression analyses on waitlist, patient, and graft survival outcomes, including their lower confidence bounds. Higher E-values support stronger evidence for the observed associations.

The E-value formula is as follows:

(1) $$E=HR+\sqrt{HR\times (HR-1)}$$

All statistical analyses were conducted using Stata version 18.0 (StataCorp LP, College Station, TX, USA).

## 3. Results

### 3.1. Cohort Characteristics

Between 2002 and 2020, our study identified 2759 patients with ALF who were waitlisted for LT. The cohort characteristics are detailed in Table 1. Across all groups, the majority of patients were Caucasian, with a higher prevalence of females. Patients with WD were significantly younger than those in other groups, with a median age of 28 years. The median ages in the HAV, HBV, and AIH groups were similar, at 45 years. Patients in the AIH group had a higher BMI compared to other groups, while those with a viral etiology were more likely to have DM (15%). Regarding waiting time for transplantation, the median time across most groups was 3 days (*p* < 0.001). However, the viral and APAP etiology groups had the shortest median waiting time of 2 days (*p* < 0.001). For further details on recipient and donor characteristics, please refer to Table 1.

### 3.2. Waitlist Outcomes

Observed outcome rates for the overall cohort were as follows: 73.8% for LT (N = 2014), 9.7% for waitlist mortality (n = 290), 8.3% for spontaneous recovery (n = 229), and 8.2% for conditions that deteriorated, making patients too sick to transplant (n = 226). In the stepwise multivariate analysis, the APAP group (HR 1.7; 95% CI, 1.29–2.22; *p* < 0.001) and the unknown etiology group (HR 1.3; 95% CI, 1.02–1.66; *p* < 0.037) had an increased risk of waitlist removal, while WD was associated with a lower risk of waitlist mortality or removal (HR 0.36; 95% CI, 0.19–0.66; *p* < 0.001). After accounting for other patient factors at waitlisting, a higher MELD score at listing (HR 1.02; 95% CI, 1.01–1.03; *p* < 0.001), elevated serum sodium (HR 1.02; 95% CI, 1.01–1.04; *p* = 0.005), and encephalopathy (HR 1.51; 95% CI, 1.26–1.89; *p* < 0.001) were found to have a detrimental impact on the risk of being removed from the waitlist. Among socioeconomic variables, patients without a college or university degree appeared to be at higher risk for poorer waitlist outcomes (HR 1.42; 95% CI, 1.19–1.7; *p* < 0.001) (Table 2).

**Figure 2:**
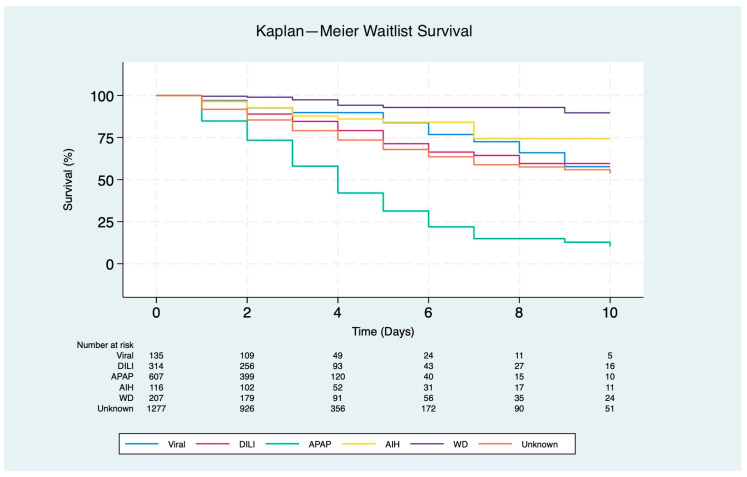
Kaplan–Meier survival curves for waitlist outcomes at 10 days in patients listed for liver transplantation due to acute liver failure (*p* < 0.001). Etiologies include viral (hepatitis A and B), drug-induced liver injury (DILI), autoimmune hepatitis (AIH), acetaminophen (APAP), Wilson disease (WD), and unknown etiology.

Patients with underlying APAP had the lowest predicted probability of receiving LT, with 84% on day 1 (*p* < 0.001), 58% on day 3 (*p* < 0.001), and 31% on day 5 (*p* < 0.001). In contrast, patients with WD had the highest overall probability of receiving LT, with 1-, 3-, and 5-day recipient survival rates of 99%, 97%, and 93%, respectively (*p* < 0.001) (Figure 2).

### 3.3. Patient Survival

From the initial cohort, 2014 patients received a LT. Our multivariate analysis showed that WD was associated with improved survival compared to HAV and HBV infections (HR 0.53; 95% CI, 0.36–0.8; *p* = 0.002) (Table 3). Another factor independently associated with improved survival was Asian race (HR 0.38; 95% CI, 0.24–0.63; *p* < 0.001). The following recipient characteristics were identified as risk factors for patient mortality: Black/African American race (HR 1.47; 95% CI, 1.21–1.88; *p* < 0.001), public insurance (HR 1.31; 95% CI, 1.1–1.57; *p* = 0.003), presence of DM (HR 1.81; 95% CI, 1.37–2.38; *p* < 0.001), and HE (HR 1.27; 95% CI, 1.06–1.52; *p* < 0.001). Additionally, donor age (HR 1.01; 95% CI, 1.00–1.02; *p* < 0.001) was associated with an increased risk of patient failure.

**Figure 3:**
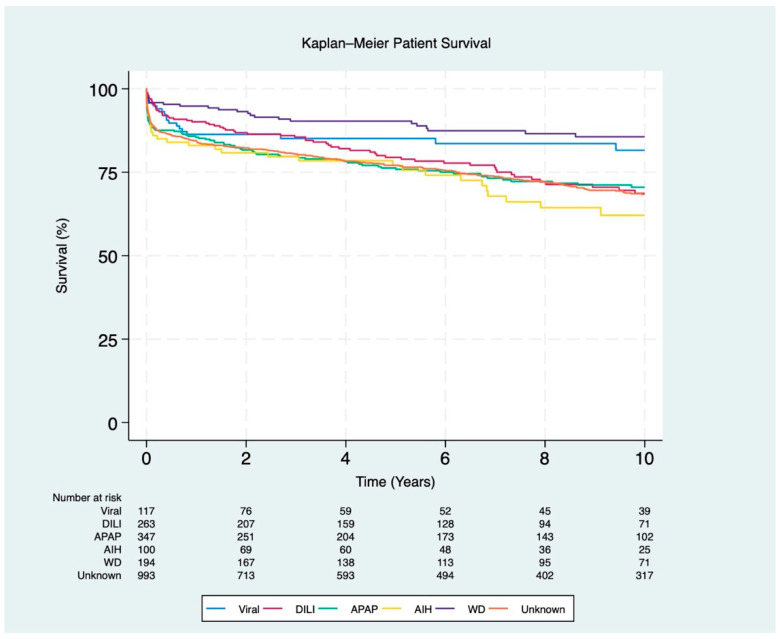
Kaplan–Meier survival curves for post-transplant patients stratified by ALF etiology (*p* < 0.001). The etiologies include viral (hepatitis A and B), DILI (drug-induced liver injury), APAP (acetaminophen), AIH (autoimmune hepatitis), WD (Wilson disease), and unknown etiology.

Post-transplant survival rates for patients with AIH were the lowest at 1 year and 10 years (83% and 62%, respectively; *p* < 0.001), while APAP had the lowest 5-year survival rate (76%; *p* < 0.001). In contrast, WD had the highest overall 1-, 5-, and 10-year recipient survival rates (95%, 90%, and 86%, respectively; *p* < 0.001) (Figure 3).

### 3.4. Graft Survival

In the multivariate analysis, Black/African American race (HR 1.56; 95% CI, 1.3–1.87; *p* < 0.001), reliance on public insurance (HR 1.24; 95% CI, 1.05–1.47; *p* = 0.012), DM (HR 1.49; 95% CI, 1.14–1.95; *p* = 0.002), and grade 3–4 encephalopathy (HR 1.36; 95% CI, 1.15–1.61; *p* < 0.001) were associated with a negative impact on post-LT graft survival. Similarly, advanced donor age (HR 1.01; 95% CI, 1.00–1.02; *p* < 0.001) and higher donor BMI (HR 1.02; 95% CI, 1.00–1.04; *p* = 0.012) were linked to an increased risk of graft failure (Table 4). WD was associated with improved survival compared to the reference group of HAV and HBV infections (HR 0.6; 95% CI, 0.42–0.86; *p* = 0.012). Additionally, Asian race (HR 0.47; 95% CI, 0.3–0.73; *p* = 0.001) was independently associated with improved graft survival.

**Figure 4:**
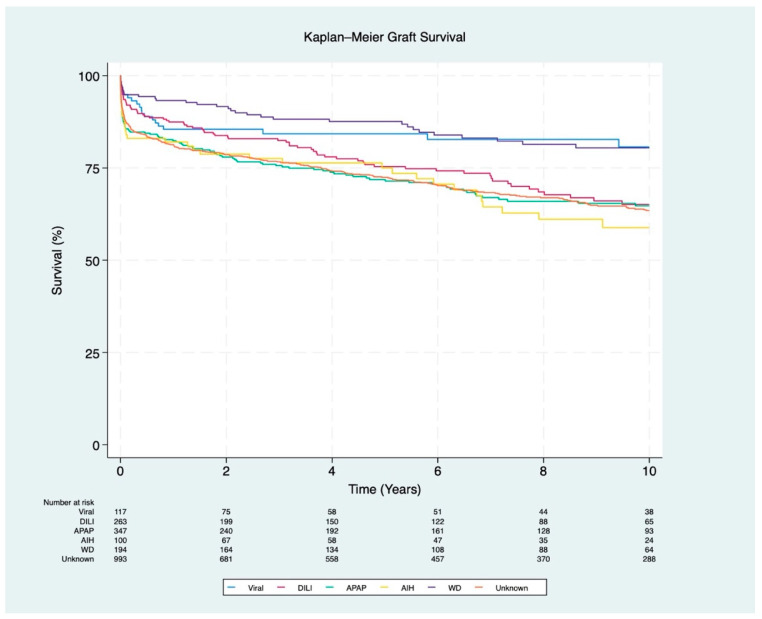
Kaplan–Meier survival curves for graft survival stratified by ALF etiology (*p* < 0.001). The etiologies include viral (hepatitis A and B), DILI (drug-induced liver injury), APAP (acetaminophen), AIH (autoimmune hepatitis), WD (Wilson disease), and unknown etiology.

Regarding graft survival, patients with AIH had the lowest 1-year and 10-year survival rates (82% and 59%, respectively; *p* < 0.001). APAP-induced liver injury was associated with the lowest 5-year survival rate (72%; *p* < 0.001). In contrast, WD had the highest overall graft survival rates, with 93% at 1 year, 88% at 5 years, and 80% at 10 years (*p* < 0.001) (Figure 4).

### 3.5. Sensitivity Analysis

In our sensitivity analysis, we calculated E-values to assess the robustness of associations with patient survival against potential unmeasured confounding. Variables such as Black race, etiology (AIH and APAP), education level (no college degree), and clinical characteristics (DM and HE) had relatively high E-values for their hazard ratios, indicating that considerable unmeasured confounding would be needed to nullify these associations. Higher E-values support the stability of these associations, even with potential confounding. Conversely, lower E-values, like those for DM and lack of a college degree, suggest some vulnerability to confounding, though moderate effects are unlikely to overturn the associations. Variables without calculated E-values indicate weak or non-positive associations with graft survival. Overall, these results reinforce the robustness of observed associations, particularly for variables with larger E-values. For further details, see Supplementary Tables S1–S3.

## 4. Discussion

Multiple factors influence outcomes in patients undergoing LT for ALF. Disease severity at listing significantly impacts waitlist outcomes, with higher MELD scores, elevated serum sodium, and encephalopathy increasing the likelihood of waitlist removal. Black/African American race, public insurance, DM, severe HE, and older donor age influence post-transplant survival. Similarly, graft survival is affected by these factors, along with higher donor BMI. Outcomes also vary by etiology, with AIH and APAP-related ALF associated with poorer prognoses. These findings underscore the need for precise risk stratification to improve management before and after LT.

Autoimmune conditions, such as AIH, are associated with poorer post-LT outcomes due to chronic progression and related complications [11]. Distinct molecular and biochemical mechanisms may underlie this association; for example, cytokines like interleukin-17 (IL-17) and tumor necrosis factor-alpha (TNF-α) are upregulated in autoimmune liver disease, driving chronic inflammation and raising graft rejection risk [12]. Additionally, oxidative stress and mitochondrial dysfunction, common in AIH, impair hepatocyte recovery and increase fibrosis risk. In severe acute cases, failure to respond within 7–14 days correlates with nearly 50% mortality. There is also growing recognition that some indeterminate acute liver failure cases may involve underlying AIH [13]. In a related analysis, Wong et al. found that AIH patients listed as Status 1 for ALF presented with high rates of ascites, likely reflecting a chronic component within the acute presentation [14].

AIH-related ALF is linked to worse post-transplant outcomes as acute AIH cases are less responsive to steroids and exhibit distinct biochemical and histological features [15]. The immune-mediated damage in AIH often results in advanced fibrosis by the time of transplantation, complicating both surgery and postoperative management [16]. Some studies suggest that corticosteroids may increase the risk of infection in AIH-ALF patients around the time of transplantation [17]. A retrospective analysis by Enke et al. involving 193 AIH-ALF cases found that 59.6% underwent transplantation by day 21, while 23.8% died before transplantation. Elevated bilirubin levels, INR, and coma grade were associated with worse outcomes, adversely affecting graft survival and overall prognosis [18].

Our study revealed that when analyzing the relationship between waitlist outcomes and the etiology of ALF, APAP-related ALF had the lowest predicted probability of receiving LT compared to other causes and the lowest five-year post-LT survival. This finding correlates with the observation that patients with APAP-related ALF have the shortest duration from waitlist to LT. A retrospective study by the ALFSG found that these patients often progress rapidly, either to death or spontaneous recovery, leading to the highest rates of waitlist removal and the lowest rates of transplantation [7].

In the U.S., the majority of liver injuries stem from APAP overdose or idiosyncratic drug reactions, many of which may be preventable. A prospective study conducted at 17 U.S. centers found that APAP overdose is the leading cause of ALF, accounting for 39% of cases, while idiosyncratic drug reactions contribute an additional 13% [19]. Short-term transplant-free survival rates for APAP-related ALF were 68%, in sharp contrast to the 17–25% survival rates associated with other drug reactions [19]. Similarly, the significant burden of ALF due to APAP injury often leads to multi-organ failure, complicating both pre- and post-LT management and increasing the risks of death and graft loss [20]. In contrast, a retrospective European Liver Transplant Registry (ELTR) study reported a steady increase in transplants for APAP-related ALF in Europe. However, 8% of these patients experienced graft loss or death due to social factors, such as suicide or non-adherence to medications, with most fatalities occurring within the first year post-transplant. These findings highlight the need for comprehensive psychological and social support for patients facing APAP-related ALF [4,21].

Patients with underlying WD showed an overall improved survival rate compared to those with other etiologies of ALF. A retrospective analysis of 515 adult WD patients, utilizing UNOS data from 1987 to 2016, reported notably higher survival rates at 3, 5, and 10 years, specifically 87.5%, 85.4%, and 80.5%, respectively, when compared to patients undergoing LT for other indications (*p* < 0.001) [22]. The higher survival rates in WD patients can be attributed to their younger demographic and lower rates of comorbidity [23]. Consistent with these findings, our study revealed that WD patients presented at a younger age and exhibited a reduced incidence of grade 3–4 HE, a critical factor since severe HE is associated with diminished post-LT survival [24]. Furthermore, the absence of disease recurrence in WD and the normalization of serum copper levels following LT may further elucidate the superior patient and graft survival rates observed in this population [25]. These findings collectively support the efficacy of LT as a viable therapeutic intervention for ALF related to WD.

The severity of encephalopathy and the presence of cerebral edema are among the most important factors influencing survival outcomes in ALF. However, the exact mechanisms and prevalence of these complications remain poorly understood [5,26]. A retrospective analysis of the UNOS database revealed that patients who underwent living donor liver transplantation (LDLT) were significantly less likely to develop ascites (*p* < 0.001) or HE (*p* = 0.04) and required less dialysis (*p* = 0.02) [27]. Patients with lower grades of encephalopathy (I or II) had a 77% survival rate, compared to 56% for those with more severe encephalopathy [20].

Regarding other independent factors associated with LT survival, our model identified DM as a significant risk factor for patient and graft survival. DM has a well-documented impact on long-term survival following LT [28]. A study using data from the SRTR, analyzing patients who underwent LT between 1987 and 2019, demonstrated that pre-existing DM, elevated creatinine, hypertension, and the use of steroids or sirolimus for immunosuppression are associated with poorer post-transplant survival [29]. Immunosuppressive agents may further worsen DM, increasing the risk of accelerated atherosclerosis and infections [30]. Early recognition of pre-DM as a risk factor for mortality can allow for optimized diabetic management during the pre-transplant period, improving patient outcomes [29].

Previous studies have identified race as a significant independent predictor of post-LT survival. Notably, African American (AA) patients have been shown to experience disproportionately poorer outcomes in both the short and long term when compared to other racial groups [31,32]. We observed a significant difference in survival between AA and white patients, with Asians having the best post-LT outcomes. Thuluvath et al., through an analysis of the UNOS liver transplant database from 1999 to 2008, found that race significantly impacts long-term graft and patient survival. AA had the poorest graft survival rates, with 60.0% at 5 years and 45.4% at 10 years, while Asians had the best outcomes, with survival rates of 88.1% at 1 year, 73.7% at 5 years, and 63.1% at 10 years [33]. The smaller body size and lower comorbidity rates in Asian patients may be contributing factors [34]. Similarly, a retrospective study by Salam et al., using UNOS data, found that AA patients had higher rates of graft failure and lower survival at 1, 3, and 5 years post-LT compared to White Caucasian patients [35]. This disparity may result from underlying conditions, delayed referrals, and limited socioeconomic and insurance support [32].

In addition to examining racial disparities, we considered socioeconomic factors, such as a lack of college education, reliance on public insurance, and non-U.S. citizenship, which may influence the group differences observed. Specifically, a lack of college education was linked to poorer waitlist outcomes, while dependence on public insurance was associated with lower survival rates for both patients and grafts. This reliance often indicates limited financial resources and other socioeconomic challenges that restrict access to quality healthcare, including timely assessments and treatments. Patients who depend on public insurance may also encounter additional barriers, such as limited transportation options and difficulties navigating the healthcare system, which can hinder access to transplant services and adherence to post-transplant care [34,36]. These obstacles likely contribute to adverse effects on both waitlist outcomes and overall survival rates [34,36,37]. Addressing these socioeconomic barriers through targeted policy changes could help reduce disparities and improve transplant outcomes for underserved populations.

Donor age is a significant risk factor for both patient and graft survival in ALF cases. Comparably, data from the UNOS [38] and the ELTR [4] have similarly demonstrated that donor and recipient ages, particularly those over 50, are associated with poorer transplant outcomes. Additionally, a single-center study including 310 adult patients with ALF listed for emergency LT found that 60% of grafts from donors older than 60 years were associated with poorer outcomes [39]. These findings mirror the global increase in donor age, an established risk factor for both elective and emergency LT [4].

An elevated donor BMI has been associated with poorer graft survival outcomes. Recent studies have similarly reported links between obesity and ALF [40]. LT evaluations frequently use BMI as a proxy for assessing hepatic steatosis [41]. However, a systematic review by Takagi et al. found no correlation between donor BMI and patient or graft survival in adult donors after brain death (DBD) or deceased donor liver transplantation (DDLT) [42]. Nevertheless, a higher donor BMI was associated with an increased risk of macrosteatosis and a significantly higher rate of liver graft rejection [42,43]. Macrosteatosis ≥30% in grafts has been identified as a risk factor for early graft dysfunction. Currently, most centers use a BMI threshold of ≥30 to 35 kg/m^2^ to exclude potential donors, as a BMI ≥35 kg/m^2^ is considered clinically significant, leading to surgical challenges, higher rates of postoperative infections, and complications with the liver graft [41,43].

The primary strength of our study is the use of an extensive, comprehensive database of LT recipients, allowing our findings to reflect nationwide trends in LT for ALF. However, several limitations must be acknowledged. First, the retrospective observational design and the dataset’s limited scope present inherent constraints. Our E-value analysis indicates that the associations found between pre-transplant waitlist outcomes and post-transplant mortality are robust against potential unmeasured confounding, at least for measured variables. Additionally, as the UNOS database includes only ALF patients listed for LT, our findings may have limited generalizability to the broader ALF population. Relying on pre-existing data also restricted control over certain variables, introducing a potential for recall bias. Despite these limitations, our study has notable strengths that enhance the credibility of our conclusions.

## 5. Conclusions

Our study highlights significant variations in post-LT outcomes based on the underlying etiologies of ALF. Patients with AIH-induced ALF had poorer outcomes, mainly due to reduced steroid responsiveness and the presence of advanced fibrosis, which complicated post-LT management. APAP liver injury showed higher mortality and graft failure rates due to factors such as multi-organ failure and psychosocial challenges. In contrast, WD patients had better post-LT survival, were aided by their younger age, had fewer comorbidities, and had no disease recurrence. Donor factors, such as age and BMI, were also significant predictors of post-LT survival. These findings emphasize the need for individualized management strategies to improve patient and graft outcomes in ALF.

## Figures and Tables

**Table 1:** Baseline cohort clinical characteristics (n = 2759).

Variable	Viral Infection HAV, HBV (n = 138)	DILI (n = 325)	APAP (n = 652)	AIH (n = 117)	Wilson Disease (n = 209)	Unknown (n = 1318)	*p* Value
recipient							
Age, (IQR)	45 (40–59)	40 (35–52)	40 (31–41)	45 (35–56)	28 (21–40)	40 (32–49)	<0.001
Female gender, n (%)	51 (37)	244 (75)	512 (79)	98 (84)	124 (59)	901 (68)	<0.001
Race, n (%)							<0.001
White Caucasian	68 (43)	176 (54)	520 (80)	48 (41)	150 (72)	750 (57)	
Black	22(16)	71 (22)	61 (9)	45 (38)	11 (5)	292 (22)	
Hispanic	12 (9)	45 (14)	45 (38)	16 (14)	34 (16)	151 (11)	
Asian	36 (26)	27 (8)	11 (5)	3 (3)	11 (5)	99 (7)	
Other	0	6 (2)	292 (22)	5 (4)	3 (1)	26 (2)	
College or university degree, n (%)	59 (43)	143 (44)	205 (31)	56 (48)	113 (54)	449 (34)	<0.001
Private insurance, n (%)	97 (70)	213 (66)	413 (63)	76 (65)	141 (68)	873 (66)	0.641
Citizenship, n (%)	125 (91)	300 (92)	634 (97)	106 (91)	194 (93)	1242 (94)	<0.001
Blood type, n (%)							
O	58 (42)	152 (47)	319 (49)	62 (53)	105 (50)	105 (47)	
A	52 (38)	105 (32)	237 (36)	31 (27)	70 (33)	70 (36)	
B	21 (15)	54 (16)	69 (11)	19 (16)	21 (10)	21 (14)	
AB	7 (5)	14 (4)	27 (4)	5 (4)	13 (6)	13 (3)	
BMI, (IQR)	27 (24–30)	27 (24–32)	25 (22–29)	28 (25–35)	27 (24–32)	27 (24–32)	<0.001
DM, n (%)	20 (15)	23 (7)	19 (3)	12 (11)	5 (2)	95 (8)	
MELD at listing, (IQR)	38 (33–44)	35 (30–40)	37 (31–43)	33 (29–37)	35 (28–41)	31 (36–41)	<0.001
Sodium, (IQR)	139 (135–140)	138 (136–141)	139 (137–142)	139 (135–141)	137 (133–141)	139 (136–141)	<0.001
INR, (IQR)	4 (2.8–5.6)	3.5 (2.6–5.1)	4.6 (3–7.3)	3.1 (2.3–4.2)	3 (2.3–4)	3.5 (2.5–5.3)	<0.001
Total bilirubin, (IQR)	20 (15–28)	21 (11–28)	4 (3–6)	23 (19–29)	24 (11–36)	18 (7–26)	<0.001
Creatinine, (IQR)	1.4 (0.8–2.6)	1 (0.7–1.9)	2 (1–3.1)	0.93 (0.67–1.5)	1.2 (0.8–2)	1.2 (0.8–2.5)	<0.001
Ascites, n (%)	74 (54)	142 (44)	195 (30)	68 (58)	153 (73)	598 (45)	<0.001
Grade 3/4 hepatic encephalopathy, n (%)	81 (59)	167 (51)	451 (69)	62 (53)	48 (23)	798 (61)	<0.001
Wait time (days), (IQR)	2 (2–4)	3 (2–4)	2 (1–3)	3 (2–6)	3 (2–6)	3 (2–4)	<0.001
donor							
Age, (IQR)	37 (25–49)	37 (25–45)	37 (36–39)	37 (25–50)	36 (23–48)	37 (25–46)	<0.001
Female Gender, n (%)	51 (44)	111 (42)	168 (48)	40 (40)	78 (40)	409 (41)	<0.001
BMI, (IQR)	26 (22–27)	26 (23–28)	26 (25–26)	25 (24–28)	26 (23–28)	25 (23–27)	<0.001
Cold ischemia time (hours), (IQR)	6 (5–7)	6 (5–7)	6 (6–7)	7 (5–7)	7 (5–8)	6 (5–7)	0.149

AIH: autoimmune hepatitis; APAP: acetaminophen; BMI: body mass index; DILI: drug-induced liver injury; DM: diabetes mellitus; HAV: hepatitis A virus; HBV: hepatitis B virus; MELD: Model for End-Stage Liver Disease. Reference ranges for sodium: 135–145 mmol/L; creatinine: 0.50–1.10 mg/dL; bilirubin: 0.3–1.2 mg/dL; INR: 0.8–1.1. The Kruskal–Wallis test was used for continuous variables, and Pearson’s chi-squared test (χ^2^) was used for categorical variables. Continuous variables were reported as medians with interquartile ranges (IQR), while categorical variables were summarized as percentages.

**Table 2:** Cox proportional hazards regression model of predictors of waitlist outcomes.

Variable	Univariate			Variable	Multivariate		
	Hazard Ratio	95% CI	*p* Value		Hazard Ratio	95% CI	*p* Value
Etiology							
Viral: HAV, HBV	[Reference]			Viral: HAV, HBV	[Reference]		
DILI	1.45	0.86–2.47	0.164	APAP	1.7	1.29–2.22	<0.001
APAP	4.56	2.84–7.39	<0.001				
AIH	0.86	0.44–1.67	0.651				
Wilson	0.33	0.16–0.69	0.003				
Unknown	1.87	1.16–3.00	0.010				
Age	0.99	0.99–1.00	0.603				
Gender, male	0.71	0.59–0.85	<0.001				
Race				Wilson	0.36	0.19–0.66	<0.001
White Caucasian	[Reference]						
Black	0.78	0.63–0.96	0.20				
Hispanic	0.6	0.45–0.79	<0.001				
Asian	0.51	0.35–0.74	<0.001	Unknown	1.3	1.02–1.66	0.037
Other	0.72	0.40–1.29	0.271				
No college or university degree	1.79	1.51–2.12	<0.001	No college or university degree	1.42	1.19–1.70	<0.001
Public insurance	1.19	1.01–1.39	0.034				
U.S. citizenship	0.71	0.50–1.02	0.551				
Blood type				MELD at listing	1.02	1.01–1.03	<0.001
O	[Reference]						
A	1.04	0.88–1.23	0.645	Serum sodium	1.02	1.01–1.04	0.005
B	0.9	0.70–1.14	0.376				
AB	0.86	0.54–1.36	0.508				
BMI	0.97	0.96–0.98	<0.001	Bilirubin	0.95	0.94–0.96	<0.001
DM	0.79	0.56–1.11	0.177				
MELD at listing	1.01	1.00–1.02	<0.001				
Serum sodium	1.05	1.03–1.06	<0.001				
INR	1.03	1.02–1.04	<0.001				
Bilirubin	0.94	0.93–0.95	<0.001				
Serum creatinine	1.14	1.10–1.18	<0.001	Encephalopathy	1.51	1.26–1.89	<0.001
Ascites	0.66	0.56–0.77	<0.001				
Encephalopathy	2.16	1.83–2.55	<0.001				

AIH: autoimmune hepatitis; APAP: acetaminophen; BMI: body mass index; CI: confidence interval; DILI: drug-induced liver injury; DM: diabetes mellitus; HAV: hepatitis A virus; HBV: hepatitis B virus; MELD: Model for End-Stage Liver Disease; U.S.: United States.

**Table 3:** Cox proportional hazards regression model of predictors of patient survival.

Variable	Univariate			Variable	Multivariate		
	Hazard Ratio	95% CI	*p* Value		Hazard Ratio	95% CI	*p* Value
recipient							
Etiology							
Viral: HAV, HBV	[Reference]			Viral: HAV, HBV	[Reference]		
DILI	1.38	0.85–2.22	0.192	Wilson	0.53	0.36–0.8	0.002
APAP	1.61	1.02–2.55	0.042				
AIH	1.92	1.13–3.28	0.017				
Wilson	0.65	0.37–1.15	0.140				
Unknown	1.55	1.00–2.39	0.047				
Age	1.00	0.99–1.00	0.199				
Gender, male	1.09	0.91–1.30	0.343				
Race							
White Caucasian	[Reference]			White Caucasian	[Reference]		
Black	1.66	1.37–2.01	<0.001	Black	1.47	1.21–1.88	<0.001
Hispanic	0.8	0.60–1.08	0.145				
Asian	0.41	0.25–0.66	<0.001	Asian	0.38	0.24–0.63	<0.001
Other	1.44	0.82–2.51	0.195				
No college or university degree	1.3	1.09–1.55	0.003				
Public insurance	1.3	1.10–1.54	0.003	Public insurance	1.31	1.1–1.57	0.003
U.S. citizenship	0.64	0.42–1.00	0.051				
Blood type							
O	[Reference]				[Reference]		
A	0.87	0.72–1.05	0.149	DM	1.81	1.37–2.38	<0.001
B	1.18	0.93–1.50	0.172				
AB	0.66	0.40–1.08	0.97				
BMI	0.99	0.99–1.00	0.997				
DM	1.91	1.46–2.51	<0.001				
Serum sodium	1.03	1.00–1.04	0.004				
MELD at listing	1.01	1.00–1.02	0.042				
INR	1.01	0.99–1.02	0.364				
Bilirubin	0.99	0.98–0.99	0.032				
Serum creatinine	1.05	1.00–1.10	0.023	Encephalopathy	1.27	1.06–1.52	<0.001
Ascites	1.04	0.88–1.23	0.640				
Encephalopathy	1.48	1.25–1.76	<0.001				
Wait time (days)	0.99	0.98–1.00	<0.001				
donor							
Age	1.01	1.00–1.02	<0.001	Age	1.01	1.00–1.02	<0.001
Gender, male	1.00	0.85–1.19	<0.001				
BMI	1.02	1.00–1.03	0.041				
Cold ischemia time	0.99	0.97–1.02	0.634				

AIH: autoimmune hepatitis; APAP: acetaminophen; BMI: body mass index; CI: confidence interval; DILI: drug-induced liver injury; DM: diabetes mellitus; HAV: hepatitis A virus; HBV: hepatitis B virus; MELD: Model for End-Stage Liver Disease; U.S.: United States.

**Table 4:** Cox proportional hazards regression model of predictors of graft survival.

Variable	Univariate			Variable	Multivariate		
	Hazard Ratio	95% CI	*p* Value		Hazard Ratio	95% CI	*p* Value
recipient							
Etiology							
Viral: HAV, HBV	[Reference]			Viral: HAV, HBV	[Reference]		
DILI	1.53	0.96–2.43	0.073	Wilson	0.6	0.42–0.86	0.005
APAP	1.86	1.19–2.90	0.006				
AIH	2.03	1.21–3.40	0.007				
Wilson	0.86	0.50–1.43	0.533				
Unknown	1.74	1.14–2.66	0.010				
Age	0.99	0.99–1.00	0.792				
Gender, male	1.06	0.89–1.25	0.496				
Race							
White Caucasian	[Reference]			White Caucasian	[Reference]		
Black	1.66	1.38–1.98	<0.001	Black	1.56	1.3–1.87	<0.001
Hispanic	0.81	0.62–1.06	0.131				
				Asian	0.47	0.3–0.73	0.001
Asian	0.48	0.31–0.72	<0.001	Other	1.86	1.14–3.04	0.013
Other	1.82	1.11–2.97	0.016				
No college or university degree	1.3	1.10–1.53	0.002				
Public insurance	1.25	1.06–1.46	0.006	Public insurance	1.24	1.05–1.47	0.012
U.S. citizenship	0.63	0.42–0.95	0.028				
Blood type							
O	[Reference]						
A	0.87	0.73–1.03	0.125	DM	1.49	1.14–1.95	0.003
B	1.19	0.96–1.49	0.114				
AB	0.71	0.46–1.11	0.140				
BMI	0.99	0.99–1.00	0.793				
DM	1.69	1.30–2.20	<0.001				
Serum sodium	1.02	1.00–1.04	0.011				
MELD at listing	1.00	0.99–1.01	0.071				
INR	1.00	0.99–1.01	0.247				
Bilirubin	0.99	0.98–0.99	0.018				
Serum creatinine	1.04	0.99–1.08	0.052	Encephalopathy	1.36	1.15–1.61	<0.001
Ascites	1.06	0.91–1.24	0.425				
Encephalopathy	1.5	1.27–1.76	<0.001				
Wait time (days)	0.99	0.99–1.00	0.509				
donor							
Age	1.01	1.01–1.02	<0.001	Age	1.01	1.00–1.02	<0.001
Gender, male	0.97	0.83–1.13	0.707				
BMI	1.02	1.01–1.03	0.001	BMI	1.02	1.00–1.04	0.012
Cold ischemia time	1.01	0.99–1.03	0.322				

AIH: autoimmune hepatitis; APAP: acetaminophen; BMI: body mass index; CI: confidence interval; DILI: drug-induced liver injury; DM: diabetes mellitus; HAV: hepatitis A virus; HBV: hepatitis B virus; MELD: Model for End-Stage Liver Disease; U.S.: United States.

## Data Availability

Data can be requested at https://unos.org/data/ (accessed on 23 May 2024).

## Supplementary Materials

The following supporting information can be downloaded at https://www.mdpi.com/article/10.3390/jcm13226642/s1, Table S1: Sensitivity analysis of waitlist survival: hazard ratios, confidence intervals, and E-Values; Table S2: Sensitivity analysis of patient survival: hazard ratios, confidence intervals, and E-Values; Table S3: Sensitivity analysis of graft survival: hazard ratios, confidence intervals, and E-Values.
