# Supplementary material for: Acute Liver Failure Etiology Determines Long-Term Outcomes in Patients Undergoing Liver Transplantation: An Analysis of the UNOS Database
Source: J Clin Med. 2024 Nov 5;13(22):6642. doi: 10.3390/jcm13226642 (PMC11594988; doi:10.3390/jcm13226642)
Supplement: Supplementary file 1 [file jcm-13-06642-s001.zip › Table S1.pdf]

Table S1. Sensitivity Analysis of Waitlist Survival: Hazard Ratios, Confidence Intervals, and E-Values

Variable	HR	95% CI (Lower Bound)	E-value (Estimate)	E-value (Lower Bound)
Etiology				
Viral: HAV, HBV		[Reference]		
DILI	1.45	0.86	2.26	-
APAP	4.56	2.84	8.59	5.13
AIH	0.86	0.44	-	-
Wilson	0.33	0.16	-	-
Unknown	1.87	1.16	3.15	1.59
Age	0.99	0.99	-	-
Gender, Male	0.71	0.59	-	-
Race				
White Caucasian		[Reference]		
Black	0.78	0.63	-	-
Hispanic	0.6	0.45	-	-
Asian	0.51	0.35	-	-
Other	0.72	0.4	-	-
No college or university degree	1.79	1.51	2.98	2.39
Public Insurance	1.19	1.01	1.67	1.11
U.S. Citizenship	0.71	0.5	-	-
Blood Type				
O		[Reference]		
A	1.04	0.88	1.24	-
B	0.9	0.7	-	-
AB	0.86	0.54	-	-
BMI	0.97	0.96	-	-

DM	0.79	0.56	-	-
MELD at Listing	1.01	1.00	1.11	-
Serum Sodium	1.05	1.03	1.28	1.21
INR	1.03	1.02	1.21	1.16
Bilirubin	0.94	0.93	-	-
Serum Creatinine	1.14	1.10	1.54	1.43
Ascites	0.66	0.56	-	-
Encephalopathy	2.16	1.83	3.74	3.06

AIH: Autoimmune Hepatitis, APAP: Acetaminophen, BMI: Body Mass Index, CI: Confidence Interval, DILI: Drug-Induced Liver Injury, DM: diabetes mellitus, HAV: Hepatitis A Virus, HBV: Hepatitis B Virus, MELD: Model for End-Stage Liver Disease, U.S.: United States. The E-value for the estimate indicates the robustness of the HR to unmeasured confounding, while the E-value for the lower confidence bound shows the confounding strength needed to nullify this bound. E-values are only calculated for HRs and bounds >1, as values ≤1 do not indicate positive associations. Higher E-values suggest greater resistance to confounding.
