# Supplementary material for: Acute Liver Failure Etiology Determines Long-Term Outcomes in Patients Undergoing Liver Transplantation: An Analysis of the UNOS Database
Source: J Clin Med. 2024 Nov 5;13(22):6642. doi: 10.3390/jcm13226642 (PMC11594988; doi:10.3390/jcm13226642)
Supplement: Supplementary file 1 [file jcm-13-06642-s001.zip › Table S2.pdf]

Table S2. Sensitivity Analysis of Patient Survival: Hazard Ratios, Confidence Intervals, and E-Values

Variable	HR	95% CI (Lower Bound)	E-value (Estimate)	E-value (Lower Bound)
		**RECIPIENT**		
Etiology				
Viral: HAV, HBV		[Reference]		
DILI	1.38	0.85	2.10	-
APAP	1.61	1.02	2.6	1.16
AIH	1.92	1.13	3.25	1.51
Wilson	0.65	0.37	-	-
Unknown	1.55	1.0	2.47	-
Age	1.00	0.99	-	-
Gender, Male	1.09	0.91	1.4	-
Race				
White Caucasian		[Reference]		
Black	1.66	1.37	2.71	2.08
Hispanic	0.8	0.6	-	-
Asian	0.41	0.25	-	-
Other	1.44	0.82	2.24	-
No college or university degree	1.3	1.1	1.92	1.43
Public Insurance	1.19	1.01	1.92	1.43
U.S. Citizenship	0.64	0.42	-	-
Blood Type				
O		[Reference]		
A	0.87	0.72	-	-
B	1.18	0.93	-	-
AB	0.66	0.40	-	-

BMI	0.99	0.99	-	-
DM	1.81	1.37	3.02	2.08
MELD at Listing	1.01	1.00	1.11	-
Serum Sodium	1.03	1.00	1.21	-
INR	1.01	0.99	1.11	-
Bilirubin	0.94	0.93	-	-
Serum Creatinine	1.05	1.00	-	-
Ascites	1.04	0.88	1.24	-
Encephalopathy	1.48	1.25	2.32	1.81
Wait time, days	0.99	0.97	-	-
		**DONOR**		
Age	1.01	1.00	1.13	-
Gender, Male	1.00	0.85	-	-
BMI	1.02	1.00	1.16	-
Cold ischemia time	0.99	0.97	-	-

AIH: Autoimmune Hepatitis, APAP: Acetaminophen, BMI: Body Mass Index, CI: Confidence Interval, DILI: Drug-Induced Liver Injury, DM: diabetes mellitus, HAV: Hepatitis A Virus, HBV: Hepatitis B Virus, MELD: Model for End-Stage Liver Disease, U.S.: United States. The E-value for the estimate indicates the robustness of the HR to unmeasured confounding, while the E-value for the lower confidence bound shows the confounding strength needed to nullify this bound. E-values are only calculated for HRs and bounds >1, as values ≤1 do not indicate positive associations. Higher E-values suggest greater resistance to confounding.
