# Supplementary material for: Acute Liver Failure Etiology Determines Long-Term Outcomes in Patients Undergoing Liver Transplantation: An Analysis of the UNOS Database
Source: J Clin Med. 2024 Nov 5;13(22):6642. doi: 10.3390/jcm13226642 (PMC11594988; doi:10.3390/jcm13226642)
Supplement: Supplementary file 1 [file jcm-13-06642-s001.zip › Table S3.pdf]

Table S3. **Sensitivity Analysis of Graft Survival: Hazard Ratios, Confidence Intervals, and E-Values**

Variable	HR	95% CI (Lower Bound)	E-value (Estimate)	E-value (Lower Bound)
		**RECIPIENT**		
Etiology				
Viral: HAV, HBV		[Reference]		
DILI	1.53	0.96	2.43	-
APAP	1.86	1.19	3.12	1.67
AIH	2.03	1.21	3.48	1.71
Wilson	0,86	0.5	-	-
Unknown	1.74	1.14	2.87	1.54
Age	0.99	0.99	-	-
Gender, Male	1.06	0.89	-	-
Race				
White Caucasian		[Reference]		
Black	1.66	1.38	2.71	2.10
Hispanic	0.81	0.62	-	-
Asian	0.48	0.31	-	-
Other	1.82	1.11	3.04	1.46
No college or university degree	1.30	1.1	1.92	1.43
Public Insurance	1.25	1.06	1.81	1.31
U.S. Citizenship	0.63	0.42	-	-
Blood Type				
O		[Reference]		
A	0.87	0.73	-	-
B	1.19	0.96	-	-
AB	0.71	0.46	-	-

BMI	0.99	0.99	-	-
DM	1.69	1.3	2.77	1.92
MELD at Listing	1.00	0.99	-	-
Serum Sodium	1.02	1.0	-	-
INR	1.00	0.99	-	-
Bilirubin	0.99	0.98	-	-
Serum Creatinine	1.04	0.99	-	-
Ascites	1.06	0.91	-	-
Encephalopathy	1.5	1.27	2.37	1.86
Wait time, days	0.99	0.99	-	-
		**DONOR**		
Age	1.01	1.01	-	-
Gender, Male	0.97	0.83	-	-
BMI	1.02	1.01	1.16	1.11
Cold ischemia time	0.99	0.97	-	-

AIH: Autoimmune Hepatitis, APAP: Acetaminophen, BMI: Body Mass Index, CI: Confidence Interval, DILI: Drug-Induced Liver Injury, DM: diabetes mellitus, HAV: Hepatitis A Virus, HBV: Hepatitis B Virus, MELD: Model for End-Stage Liver Disease, U.S.: United States E-value for the estimate indicates robustness of the HR to unmeasured confounding, while E-value for the lower confidence bound shows the confounding strength needed to nullify this bound. E-values are only calculated for HRs and bounds >1, as values ≤1 do not indicate positive associations. Higher E-values suggest greater resistance to confounding.
